# Transcriptome and co-expression network analysis reveals the molecular mechanism of inosine monophosphate-specific deposition in chicken muscle

**DOI:** 10.3389/fphys.2023.1199311

**Published:** 2023-05-17

**Authors:** Baojun Yu, Zhengyun Cai, Jiamin Liu, Wei Zhao, Xi Fu, Yaling Gu, Juan Zhang

**Affiliations:** College of Agriculture, Ningxia University, Yinchuan, China

**Keywords:** Jingyuan chicken, muscle, inosine monophosphate, transcriptome, weighted gene co-expression network analysis

## Abstract

The inosine monophosphate (IMP) content in chicken meat is closely related to muscle quality and is an important factor affecting meat flavor. However, the molecular regulatory mechanisms underlying the IMP-specific deposition in muscle remain unclear. This study performed transcriptome analysis of muscle tissues from different parts, feeding methods, sexes, and breeds of 180-day-old Jingyuan chickens, combined with differential expression and weighted gene co-expression network analysis (WGCNA), to identify the functional genes that regulate IMP deposition. Out of the four comparison groups, 1,775, 409, 102, and 60 differentially expressed genes (DEGs) were identified, of which *PDHA2*, *ACSS2*, *PGAM1*, *GAPDH*, *PGM1*, *GPI*, and *TPI1* may be involved in the anabolic process of muscle IMP in the form of energy metabolism or amino acid metabolism. WGCNA identified 11 biofunctional modules associated with IMP deposition. The brown, midnight blue, red, and yellow modules were strongly correlated with IMP and cooking loss (*p* < 0.05). Functional enrichment analysis showed that glycolysis/gluconeogenesis, arginine and proline metabolism, and pyruvate metabolism, regulated by *PYCR1*, *SMOX*, and *ACSS2*, were necessary for muscle IMP-specific deposition. In addition, combined analyses of DEGs and four WGCNA modules identified *TGIF1* and *THBS1* as potential candidate genes affecting IMP deposition in muscle. This study explored the functional genes that regulate muscle development and IMP synthesis from multiple perspectives, providing an important theoretical basis for improving the meat quality and molecular breeding of Jingyuan chickens.

## 1 Introduction

With further economic and societal developments, the demand for meat products continues to increase, and more focus is being placed on meat quality. The second most consumed meat in China is poultry, and its demand is increasing annually. It has become an essential source of meat in daily life. In recent years, there has been a significant improvement in meat production through the use of modern specialized broiler chickens. This improvement can be attributed to high-intensity selection for growth rate in poultry breeding efforts (Maharjan et al., 2021). However, the quality of chicken meat has significantly decreased, particularly meat flavor (Bao et al., 2008; Katemala et al., 2021). Therefore, while improving poultry growth rate, cultivating high-quality local chicken breeds with excellent meat quality and unique flavors has become the main research direction for modern poultry molecular breeding.

Animal muscle quality includes various indicators, such as pH, water-holding capacity (WHC), cooking loss, shear force, and flavor. Muscle flavor is an important aspect of meat quality evaluation, and mainly includes umami and aroma (Liu et al., 2017). Inosine monophosphate (IMP) is the most important umami substance in livestock and poultry muscles, and the content of IMP in chicken meat is 4.5 times higher than that of free glutamic acid (Ninomiya, 1998). IMP is an important precursor of meat aromas (Cambero et al., 1992). The influence of IMP on muscles is crucial for meat flavor. Muscle IMP synthesis and metabolic processes involve gene expression, signal transduction, and network regulation; however, many regulatory factors still need to be explored.

Transcriptomics can be used to identify differentially expressed genes associated with target traits from a large amount of genomic information, thus revealing the internal association between gene expression and specific physiological processes (Wang et al., 2009). However, many differentially expressed genes are typically identified in high-throughput sequencing results, and single-gene analysis frequently misses important information. Therefore, the weighted gene co-expression network analysis (WGCNA) method was developed based on global gene-expression patterns. WGCNA has been successfully used to identify gene expression networks and biomarkers of interest in multiple groups of samples (Li et al., 2018; Cheng et al., 2020; Yuan et al., 2020; Yuan and Lu, 2021), thereby alleviating the problem of multiple detections in extensive data analysis. The application of WGCNA to chicken muscle IMP deposition-related gene expression has yet to be reported.

The Jingyuan chicken is an outstanding excellent local breed that has received national recognition for its excellent quality. Known for its slow growth rate and strong resistance, this breed produces meat that is rich in amino and fatty acids, high in muscle IMP content, and incredibly delicious (Yu et al., 2022). This is an ideal animal model for studying the meat quality of slow-growing chickens. Our previous studies have revealed differences in IMP content among Jingyuan chicken parts, feeding methods, sex, and breeds (Zhang et al., 2020; Zhang et al., 2021; Wang et al., 2022). Therefore, in this study, transcriptome sequencing was performed on breast and leg muscles of caged Jingyuan hens, breast muscles of caged Jingyuan roosters, and breast muscles of free-range Jingyuan chickens and Pudong hens (slow-growing local native chickens with excellent meat quality). Multiple differentially expressed genes (DEGs) and metabolic pathways closely related to IMP anabolic processes were identified in the different comparative groups. WGCNA was used to construct co-expressed gene modules related to muscle quality indicators, and *PYCR1*, *SMOX*, *ACSS2*, *TGIF1*, and *THBS1* were identified as potential candidate genes regulating IMP deposition by mRNA-trait association analysis. This study explored the molecular markers regulating muscle IMP synthesis and metabolism in Jingyuan chickens from multiple perspectives, which are economically important for improving the meat quality and molecular breeding of Jingyuan chickens and also provide an important reference for the development and utilization of local chicken breeds.

## 2 Materials and methods

### 2.1 Animal and sample collection

Jingyuan chickens were used in this study after 11 generations of purification and rejuvenation and a family-equivalent breeding population was established. Breeding was performed using the closed herd breeding method. To date, two generations of breeding conservation work have been conducted, and all experimental samples were collected at the Jingyuan Chicken National Conservation Farm (Pengyang County, Ningxia). We obtained permission to use Jingyuan chickens and fed them with the same diet as that used on the source farm. After growing to 180 days old, 15 caged Jingyuan roosters and hens, 15 free-range Jingyuan hens, and five free-range Pudong hens were randomly selected. Caged chickens were fed individually in 40 × 40 × 40 cm cages. After fasting for 12 h, the birds were killed under carbon dioxide anesthesia (inhaled 40%). Samples of breast and leg muscles were rapidly collected; one tissue sample was used for the determination of IMP, inosine, and various physical indices (Zhang et al., 2020; Zhang et al., 2021; Wang et al., 2022) (Supplementary Table S1), and another was snap-frozen in liquid nitrogen and stored at −80°C.

### 2.2 Library construction and transcriptome sequencing

The transcriptome analysis sample grouping information is shown in Table 1. Muscle tissues with different IMP contents between the groups were selected as sequencing samples. Magnetic beads with oligo (dT) were used to enrich the mRNA after assessing the total RNA quality of the muscle tissue. Purified mRNA was used as a template to synthesize single-stranded cDNA using a random hexamer primer, followed by the addition of DNA polymerase I and RNase H to synthesize double-stranded cDNA. The final cDNA library was amplified using PCR. Library quality was evaluated using the Agilent Bioanalyzer 2,100 system (Agilent Technologies, Santa Clara, CA, United States). After passing the library inspection, 15 libraries were submitted to the Illumina HiSeq platform for sequencing and completed by Beijing Nuohe Zhiyuan Technology Co., Ltd. (Beijing, China).

**TABLE 1:** Transcriptome sequencing sample grouping information.

Group	Sample name	Gender	Muscle	Feeding method	Breed
PJFXL	l27tho, l29tho, l30tho	hen	breast	caged	Jingyuan chicken
PJFTL	l17leg, l18leg, l20leg	hen	leg	caged	
PJMXL	l2tho, l6tho, l10tho	rooster	breast	caged	
PJFXS	PYs2tho, PYs3tho, PYs12tho	hen	breast	free-ranged	
JHFXS	sh3tho, sh4tho, sh5tho	hen	breast	free-ranged	Pudong chicken

### 2.3 Differential expression analysis

Raw reads were filtered after sequencing and more than 38,940,706 high-quality clean reads were obtained from each muscle sample. The mapping rate of these reads to the *Gallus gallus* reference genome was >72.14% (Supplementary Table S2). HTSeq (V0.6.1) software was used to analyze gene expression levels in 15 samples, and the FPKM of each gene was calculated (the sum of the FPKM of three replicates was less than 0.1 and can be considered not expressed). The DESeq2 (Love et al., 2014) package was used to perform differential expression analysis between groups, with *p* < 0.05 and |log_2_ (fold change)| > 1 as thresholds to identify DEGs between groups.

### 2.4 Weighted gene co-expression network analysis

WGCNA is suitable for the analysis of complex trait data and allows for further exploration of key genes affecting the phenotype. In this study, we used FPKM values obtained from mRNA-seq, removed genes with a mean FPKM <1, and applied the WGCNA package tool (Langfelder and Horvath, 2008) to construct co-expression networks. First, the Pearson correlation between genes was calculated to construct a gene co-expression correlation matrix. Subsequently, the optimal soft threshold (β = 12) was selected according to the criterion of an approximately scale-free topology, and a weighted adjacency matrix was generated. Furthermore, the adjacency matrix was converted into a topological overlap matrix (TOM) using a correlation expression value analysis. Next, the TOM was used to cluster the genes, and the clustered modules were classified using dynamic shearing to identify highly co-expressed gene modules. The key modules of interest were identified based on the first principal component computation module eigengene (ME) of the expression profile and correlated with phenotypic traits. In addition, Pearson correlations between gene expression profiles and phenotypic traits were calculated to estimate gene significance (GS). Finally, the relationship between genes and traits was quantified using the GS module. Then, we analyzed the correlation between GS and module membership (MM), and genes with GS > 0.5 and MM > 0.9 in the trait-specificity module were identified as hub genes.

### 2.5 DEGs and hub genes functional enrichment analysis

To explore the functions of the DEGs and significant modules, GO function and KEGG pathway enrichment analyses were performed using the Cluster Profiler tool. GO terms and KEGG pathways attaining *p* < 0.05 were significantly enriched for the DEGs and hub genes.

### 2.6 Key gene identification and correlation analysis

The Upset tool was used to analyze the intersection of DEGs and hub genes, and the Pearson correlation between the intersection of genes with IMP and cooking loss was calculated. The protein interaction network of the differential genes was analyzed using the STRING database (http://string-db.org/), where the minimum required interaction score was set to high confidence (0.700).

### 2.7 Real-time fluorescence quantitative PCR

The heart, liver, spleen, lungs, kidneys, abdominal fat, breast muscles, and leg muscles of 180-day-old Jingyuan chickens were collected for gene expression profiling. Total RNA was extracted using RNAiso Plus (Takara, Dalian, China) according to the manufacturer’s instructions and cDNA was synthesized using the PrimeScript RT Reagent Kit (Perfect Real Time; Takara, Dalian, China). Primer 6.0 was used to design the mRNA primers (Supplementary Table S3). qPCR was performed using a CFX96 real-time PCR detection system (Bio-Rad) according to the instructions for the SYBR^@^ Green Premix Pro Taq HS qPCR Kit (Accurate Biology, Changsha, China), with β-actin as an internal reference, and three replicates for each sample. The relative expression of mRNA was calculated using the 2^−ΔΔCt^ method, and the results are expressed as the mean ± standard deviation.

## 3 Results

### 3.1 Sequencing data evaluation

In this study, 15 mRNA-sequencing libraries were constructed for five groups of muscle samples. A total of 18,931 genes were detected (Supplementary Table S4), of which 15,947 genes were expressed in the PJFXL group, 16,326 in the PJFTL group, 15,786 in the PJMXL group, 15,717 in the PJFXS group, and 15,676 in the JHFXS group. The most abundantly expressed genes were actin alpha 1 (*ACTA1*), troponin I2, fast skeletal type (*TNNI2*), glyceraldehyde-3-phosphate dehydrogenase (*GAPDH*), myosin light chain 1 (*MYL1*), and phosphoglycerate mutase 1 (*PGAM1*).

**FIGURE 1:**
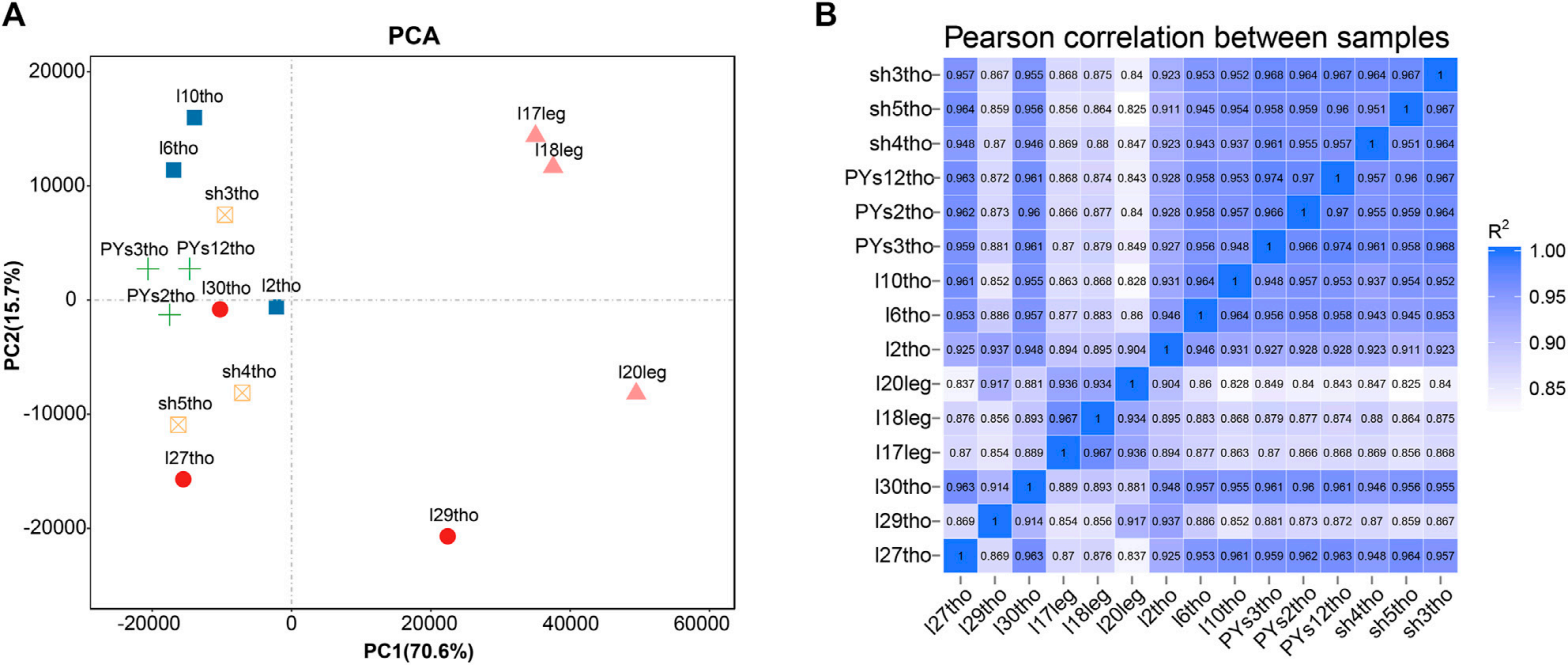
Sample principal components and correlation analysis. **(A)** Principal component analysis (PCA) of five groups of muscle samples. **(B)** Heatmap of Pearson correlation between samples based on gene expression.

Principal component analysis (PCA) and intersample correlation analyses showed that the difference between the breast and leg muscles was the most significant (PC1), with significant differences among the PJFXL, PJFXS, and JHFXS groups (Figure 1A). The Pearson correlation coefficient among samples was greater than 0.825 (Figure 1B). This indicated that the transcriptome sequencing results were reliable and could be used for subsequent analyses.

### 3.2 Screening and identification of DEGs

**FIGURE 2:**
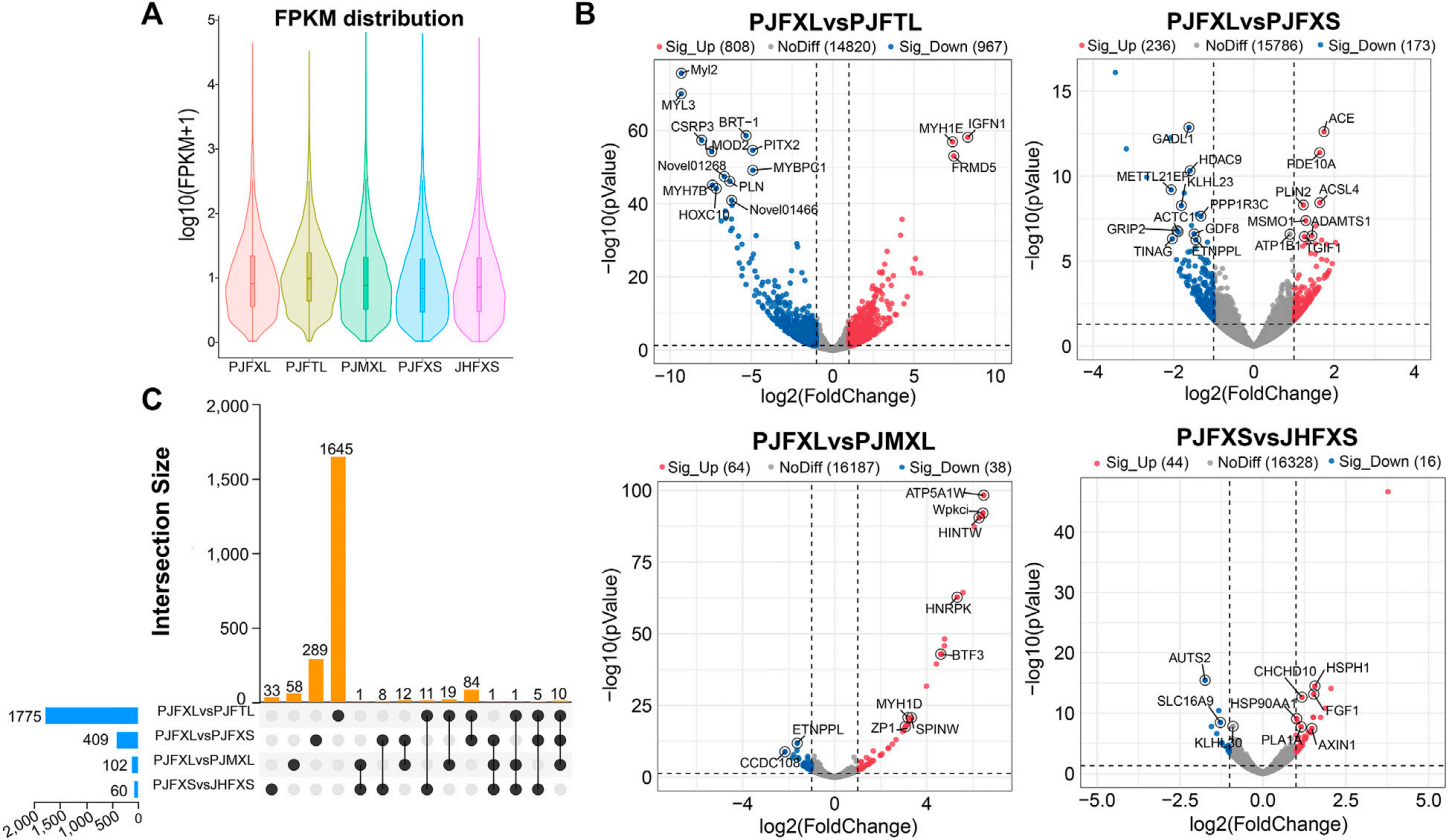
Identification of DEGs in five groups of muscle samples. **(A)** Distribution of gene expression levels in different groups. **(B)** Volcano plots of DEGs in the PJFXL vs. PJFTL, PJFXL vs. PJFXS, PJFXL vs. PJMXL, and PJFXS vs. JHFXS groups (Sig_Up: significantly upregulated; Sig_Up: significantly downregulated; NoDiff: no significant difference). **(C)** Upset diagram of four group comparatives DEGs (The horizontal bars on the left indicate the number of DEGs required for each group comparison. The individual points in the middle matrix represent the DEGs specific to each group comparison, and the lines between points represent the DEGs common to different group comparisons. The vertical bars represent the number of DEGs specific to or common to different group comparisons).

ptIn each group, 6.33%–7.59% of genes were extremely highly expressed, and the expression levels of most other genes were relatively uniform (Figure 2A). We further analyzed the DEGs between different comparison groups to investigate the key mRNAs regulating IMP-specific deposition in Jingyuan chicken muscle. We identified 1775 DEGs in the PJFXL *versus* PJFTL comparison, of which 808 were upregulated and 967 were downregulated. A total of 409 DEGs were identified in the PJFXL *versus* PJFXS comparison, of which 236 were upregulated and 173 were downregulated. A total of 102 DEGs were identified in the PJFXL *versus* PJMXL comparison, of which 64 were upregulated and 38 were downregulated. Sixty DEGs were identified in the PJFXS *versus* JHFXS comparison, of which 44 were upregulated and 16 were downregulated (Figure 2B). Hierarchical cluster analysis revealed significant differences in the expression levels of the DEGs among the comparison groups (Supplementary Figure S1). None of the DEGs were common to all four comparison groups, and there were at most ten DEGs in common among any three groups (Figure 2C).

### 3.3 GO and KEGG enrichment analysis of DEGs

**FIGURE 3:**
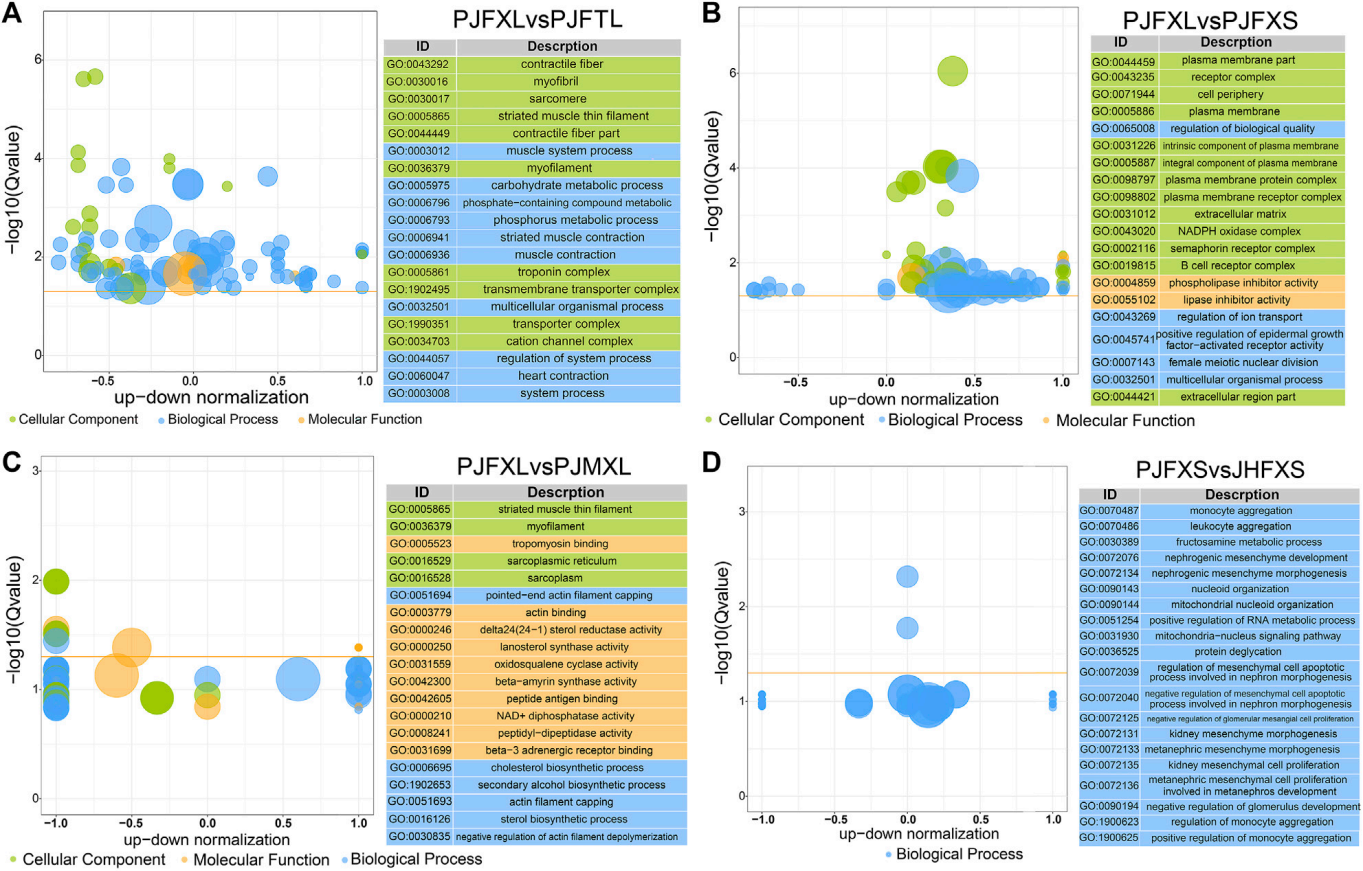
GO functional enrichment results of DEGs in **(A)** PJFXL vs. PJFTL, **(B)** PJFXL vs. PJFXS, **(C)** PJFXL vs. PJMXL, and **(D)** PJFXS vs. JHFXS group comparisons (The vertical coordinate is −log_10_ (Qvalue), the horizontal coordinate is the up-down normalization value: the difference between the number of differentially up-regulated genes and the number of differentially down-regulated genes as a percentage of the total differential genes, and the size of the bubble indicates the number of target genes currently enriched by the GO term).

To gain insight into the functions of the DEGs, we performed GO and KEGG enrichment analyses to reveal the molecular mechanism of IMP anabolism in Jingyuan chicken muscles. GO enrichment analysis showed significantly enriched DEGs in the PJFXL vs. PJFTL comparison in functional terms, such as myofibril, muscle system process, carbohydrate metabolic process, actin binding, and kinase activity (Figure 3A). The DEGs in the PJFXL vs. PJFXS comparison were mainly related to GO terms such as receptor complex, lipase inhibitor activity, and positive regulation of epidermal growth factor-activated receptor activity (Figure 3B). Twelve GO terms were significantly enriched in DEGs in the PJFXL vs. PJMXL comparison, including striated muscle thin filaments, tropomyosin binding, and pointed-end actin filament capping (Figure 3C). Most of the DEGs in the PJFXS vs. JHFXS comparison were enriched in biological processes, and the two significantly enriched GO terms were monocyte and leukocyte aggregation (Figure 3D).

**FIGURE 4:**
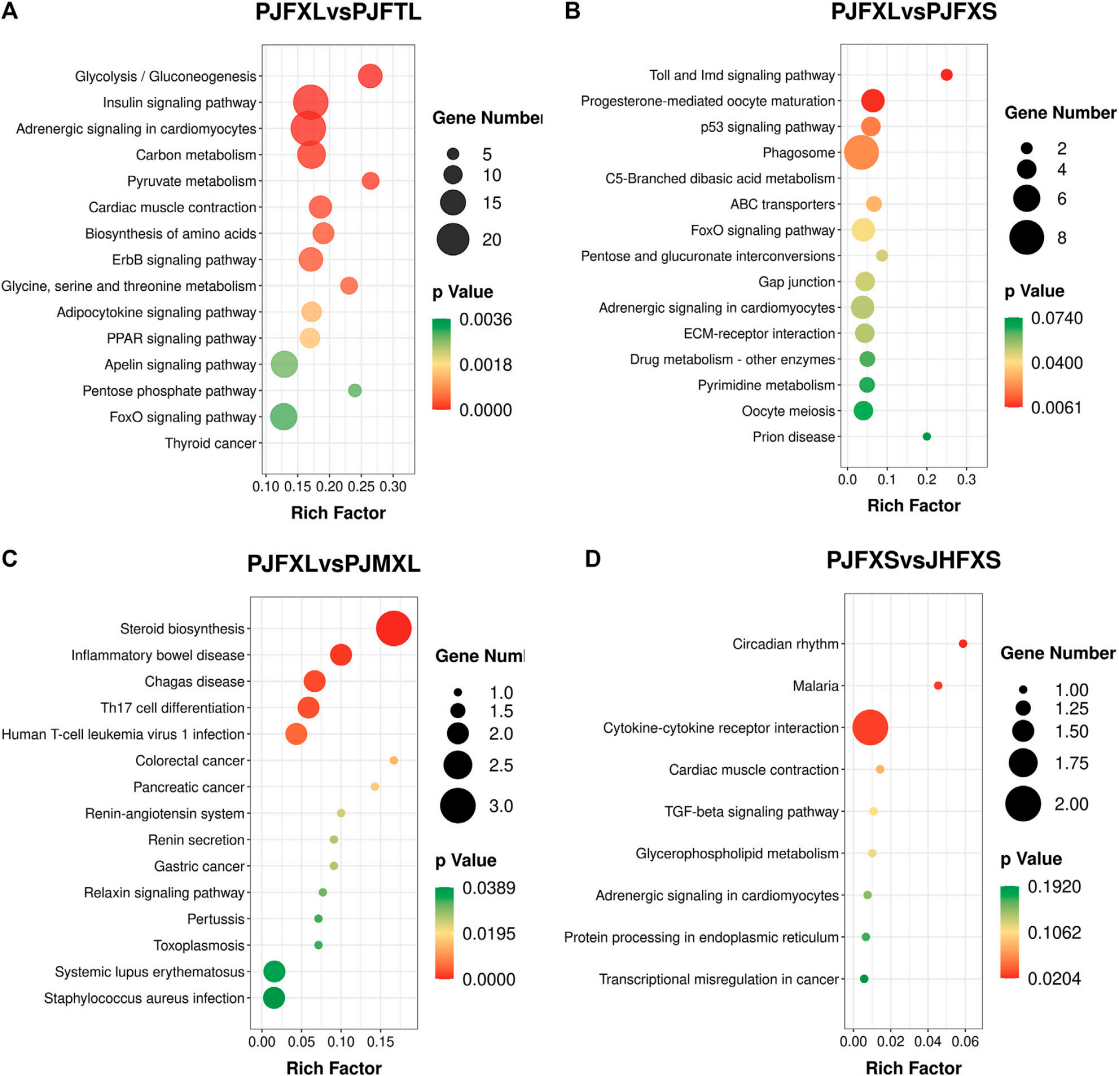
KEGG pathway enrichment results of DEGs in **(A)** PJFXL vs. PJFTL, **(B)** PJFXL vs. PJFXS, **(C)** PJFXL vs. PJMXL, and **(D)** PJFXS vs. JHFXS group comparisons (Rich Factor indicates the ratio of the number of differential genes to the number of all genes in the background gene set that are enriched in the pathway).

KEGG pathway enrichment analysis showed that DEGs in the PJFXL vs. PJFTL comparison were significantly enriched in 32 signaling pathways, including glycolysis/gluconeogenesis, carbon metabolism, pyruvate metabolism, biosynthesis of amino acids, the PPAR signaling pathway, and the FoxO signaling pathway (Figure 4A), which regulate multiple genes related to muscle development and IMP anabolism. Nine signal pathways were significantly enriched by DEGs in the PJFXL vs. PJFXS comparison, including the p53 signaling pathway, the FoxO signaling pathway, and pentose and glucuronate interconversions are essential to regulate muscle development (Figure 4B). DEGs in the PJFXL vs. PJMXL comparison were significantly enriched in 17 signaling pathways, including steroid biosynthesis and the renin-angiotensin system (Figure 4C). DEGs in the PJFXS vs. JHFXS comparison were enriched in only nine signaling pathways, including circadian rhythm, malaria, and cytokine-cytokine receptor interaction (Figure 4D). Comprehensive analysis identified *PDHA2*, *ACSS2*, *PGAM1*, *GAPDH*, *PGM1*, *GPI*, and *TPI1* as key genes regulating IMP deposition (Table 2), which are likely to be involved in muscle IMP synthesis and metabolism through a variety of mechanisms, such as energy and amino acid synthesis.

**TABLE 2:** Key pathways for significantly enriched of DEGs.

Group	Pathway	Pathway id	*p*-value	Genes
PJFXL vs. PJFTL	Glycolysis/Gluconeogenesis	ko00010	1.72E-06	*PKLR, MINPP1, PDHA2, PGK2, GPI, PGAM1, TPI1, FBP2, PGM1, BPGM, ACSS2, GAPDH, LDHA*
	Pyruvate metabolism	ko00620	0.000125	*ACSS2, PKLR, LDHA, ACYP2, ACYP1, PDHA2, GLO1, ME1*
	Biosynthesis of amino acids	ko01230	0.000299	*CYLY, TPI1, PKLR, PRPS1L1, BCAT1, GAPDH, PSPH, CTH, RPIA, PGK2, CPS1, ARF6, PGAM1*
	Adipocytokine signaling pathway	ko04920	0.001304	*SLC2A1, MAPK10, ACSBG2, PRKAG3, PRKAA2, CD36, ADIPOQ, SOCS3, ACSBG1*
	PPAR signaling pathway	ko03320	0.001487	*FABP6, LPL, CPT2, ACSBG1, ADIPOQ, ACADL, CD36, PLIN1, ACSBG2, GK2, ME1*
	Pentose phosphate pathway	ko00030	0.002986	*FBP2, PGM1, PRPS1L1, RPIA, FBP1, GPI*
	FoxO signaling pathway	ko04068	0.003087	*CDKN1A, MAP2K2, IL10, FOXO3, HOMER2, SETD7, FOXO1, PRKAG3, PIK3R3, CCND1, PRKAA2*
	Focal adhesion	ko04510	0.015515	*TLN2, CHAD, SHC2, PAK1, MYL10, THBS1, VEGFA, MYL2, CCND1, MAPK10*
	Glycerophospholipid metabolism	ko00564	0.034045	*GPD2, MBOAT2, GPAM, PISD, PLA2G2E, GPD1L, PEMT, DGKH, AGPAT2*
	Regulation of actin cytoskeleton	ko04810	0.049843	*MYL2, ENAH, PPP1CC, PIK3R3, FGF4, PAK5, GIT1, FGF9, PDGFA, PAK1, SSH2*
PJFXL vs. PJFXS	p53 signaling pathway	ko04115	0.019724	*RRM2, THBS1, CDK1, CCNB1*
	FoxO signaling pathway	ko04068	0.041711	*PLK3, CCNB3, PLK1, FOXO1, CCNB1*
	Pentose and glucuronate interconversions	ko00040	0.047352	*AKR1B10, KL*
PJFXL vs. PJMXL	Steroid biosynthesis	ko00100	9.24E-06	*LSS, MSMO1, DHCR24*
PJFXS vs. JHFXS	Cytokine-cytokine receptor interaction	ko04060	0.027194	*BMP7, THPO*

### 3.4 Weighted gene co-expression network construction

**FIGURE 5:**
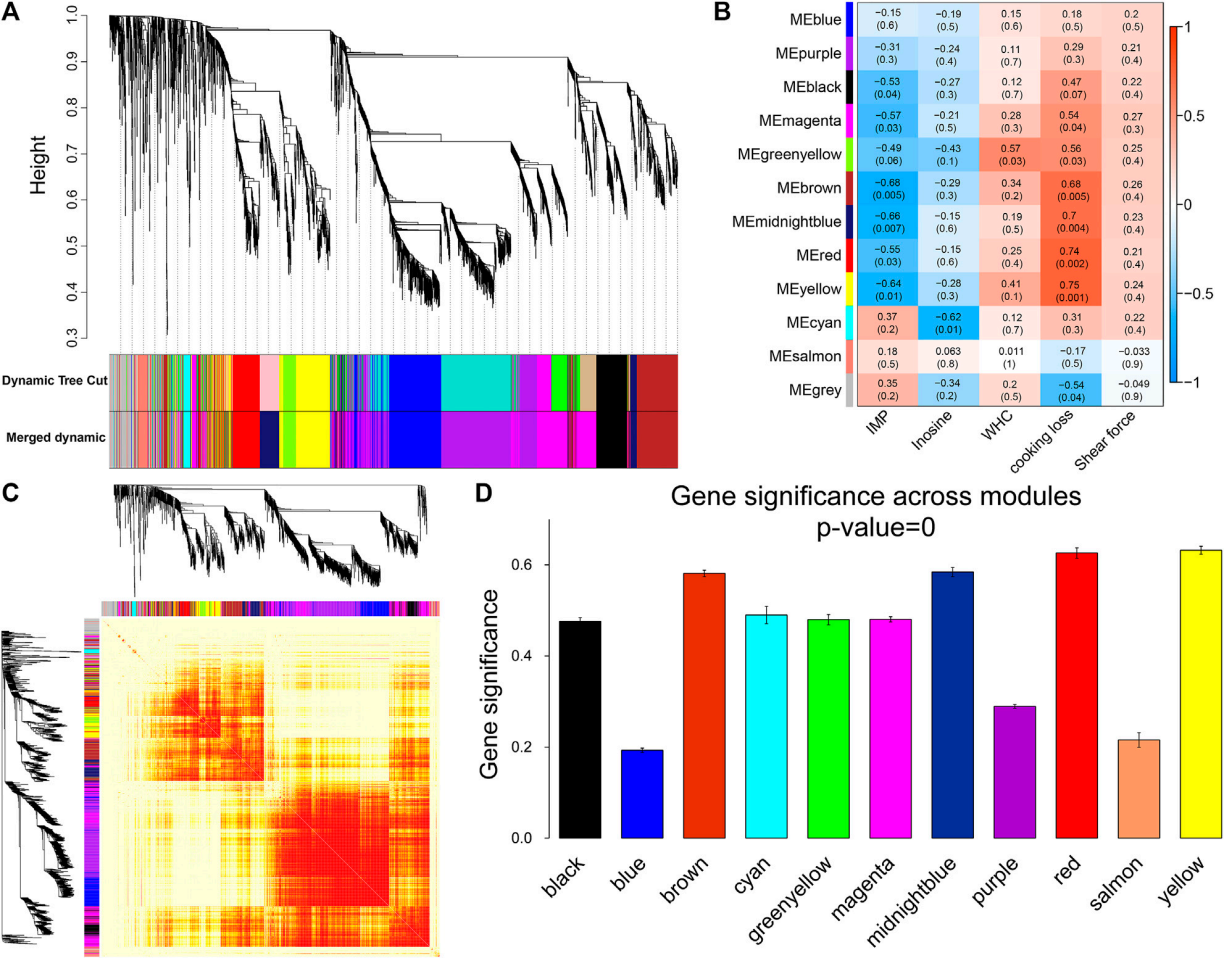
Module identification and trait correlation analysis. **(A)** Gene modules based on phylogenetic clustering tree. **(B)** Correlation analysis of 12 modules and muscle phenotypes. **(C)** Heatmap of gene co-expression network (the heatmap area indicates the dissimilarity between genes, the smaller the value, the darker the color). **(D)** Gene significance in the module.

For WGCNA analysis, genes with an average FPKM of less than 1.0 were removed. Based on the gene clustering tree analysis, the 11,640 genes were divided into 12 modules (the gray module in Figure 5A indicates that these genes had a low pattern of variation throughout the experiment and that the pattern of variation could not be used to associate with other genes; therefore, the subsequent analysis was removed). The two largest modules in Figure 5A contained 692 and 536 genes, and the two smallest modules contained 76 and 53 genes, respectively (Supplementary Table S5). A heat map of gene co-expression networks was used to explore the interactions between modules, and multiple modules were found to be interrelated (Figure 5C).

Next, the gene modules significantly associated with meat quality phenotypes were identified. We found that six modules (Figure 5B) were significantly and negatively correlated with IMP, including brown (*r* = −0.68, *p* = 0.005) and midnight blue (*r* = −0.66, *p* = 0.007). Inosine was significantly negatively correlated with the cyan module (*r* = −0.62, *p* = 0.01). Six modules (yellow, red, midnight blue, brown, green-yellow, and magenta) were positively correlated with cooking loss. The modules with strong correlations were yellow (*r* = 0.75, *p* = 0.001), red (*r* = 0.74, *p* = 0.002) and midnight blue (*r* = 0.7, *p* = 0.004). There was a significant positive correlation between WHC and the green-yellow module (*r* = 0.57, *p* = 0.03). At the same time, we found that the brown, midnight blue, red, and yellow modules were strongly correlated with both IMP and cooking loss, and the GS of these four modules was the highest among all the co-expression modules (Figure 5D).

### 3.5 Hub gene screening and functional analysis

**FIGURE 6:**
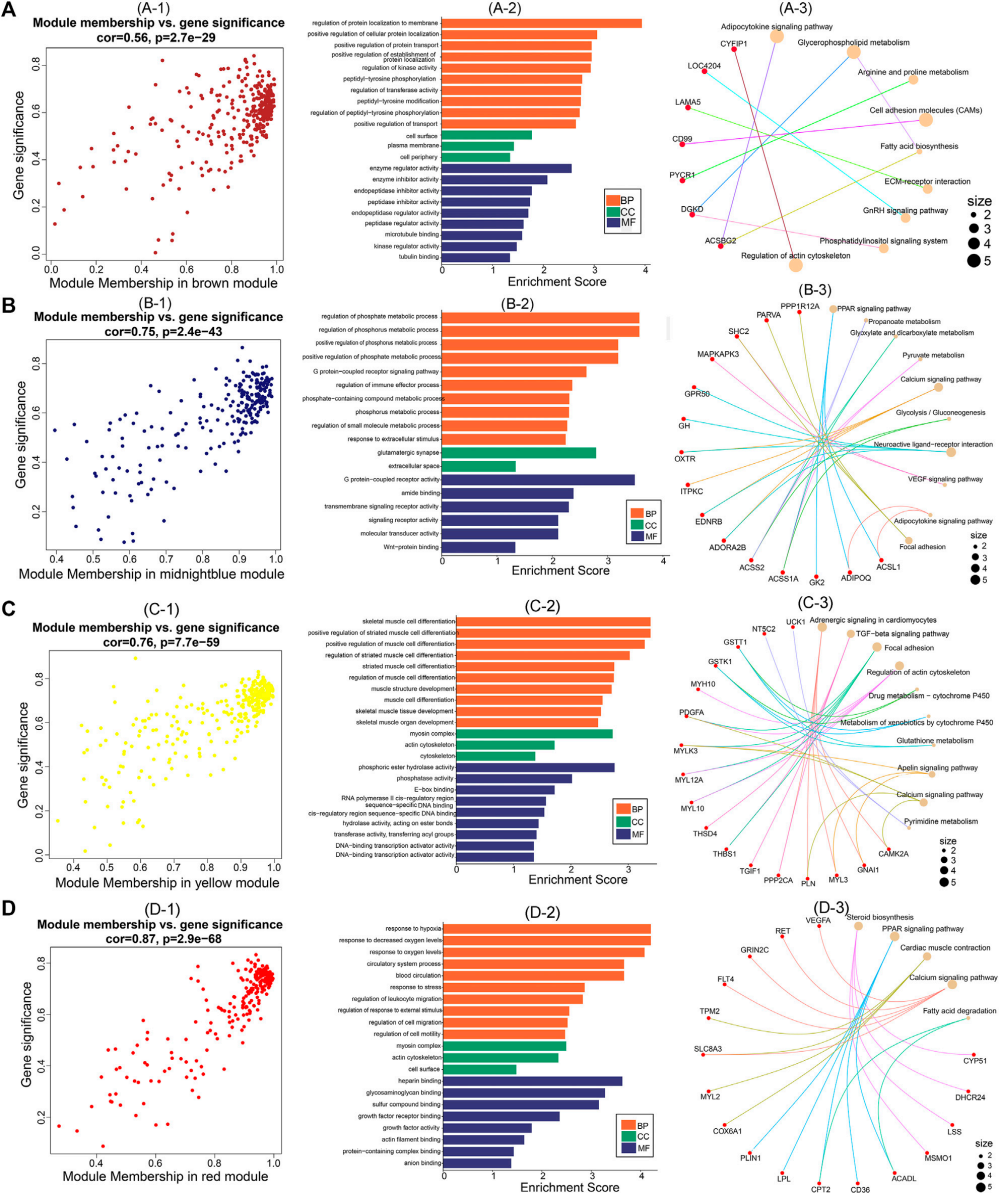
Hub genes screening and functional analysis in brown, midnightblue, red, and yellow modules. Scatter plot of gene significance and module membership for **(A)**-1 (brown), **(B)**-1 (midnightblue), **(C)**-1 (yellow), and **(D)**-1 (red) modules. GO functional enrichment analysis (BP: biological processes; CC: cellular components; MF: molecular functions) of hub genes in **(A)**-2 (brown), **(B)**-2 (midnightblue), **(C)**-2 (yellow), and **(D)**-2 (red) modules. KEGG pathway enrichment analysis of hub genes in **(A)**-3 (brown), **(B)**-3 (midnightblue), **(C)**-3 (yellow), and **(D)**-3 (red) modules (red nodes are key genes enriched in the pathway).

After identifying the four important modules, we further explored the hub genes in each module using GS and MM. The brown module obtained 178 hub genes, and the correlation between GS and MM was 0.56 (*p* = 2.7e-29) (Figures 6A–1). Functional enrichment analysis showed that hub genes in this module were significantly enriched in GO terms such as regulation of protein localization to the membrane and regulation of kinase activity (Figures 6A–2). In addition, hub genes were significantly enriched in the adipocytokine signaling, glycerophospholipid metabolism, and arginine and proline metabolism pathways (Figures 6A–3). The midnight blue module obtained 132 hub genes, and the correlation between GS and MM was 0.75 (*p* = 2.4e-43) (Figures 6B–1). Hub genes were significantly enriched in such GO terms as phosphate metabolic process regulation, small molecule metabolic process, and signaling receptor activity (Figures 6B–2). Among the nine KEGG pathways that were significantly enriched, the PPAR signaling pathway, pyruvate metabolism, and glycolysis/gluconeogenesis are necessary for muscle IMP deposition (Figures 6B–3). The yellow module obtained 177 hub genes, and the correlation between GS and MM was 0.76 (*p* = 7.7e-59) (Figures 6C–1). The hub genes in this module were significantly enriched in GO terms such as skeletal muscle cell differentiation and phosphoric ester hydrolase activity (Figures 6C–2). Twelve KEGG pathways were significantly enriched, including the TGF-β signaling pathway, regulation of the actin cytoskeleton, and glycerolipid metabolism (Figures 6C–3). The red module obtained 129 hub genes, and the correlation between GS and MM was 0.87 (*p* = 2.9e-68) (Figures 6D–1). The hub genes in this module were significantly enriched in GO terms such as actin filament binding, growth factor activity, and myosin complex (Figures 6D–2), and significantly enriched in steroid biosynthesis, PPAR signaling pathways, cardiac muscle contraction, calcium signaling pathways, and fatty acid degradation signal pathways (Figures 6D–3).

Joint differential expression analysis identified glycolysis/gluconeogenesis, arginine and proline metabolism, PPAR signaling pathway, and pyruvate metabolism as the functional pathways regulating IMP anabolism. The key genes *PYCR1*, *SMOX*, and *ACSS2* may play important regulatory roles in muscle IMP deposition.

### 3.6 Functional gene identification

**FIGURE 7:**
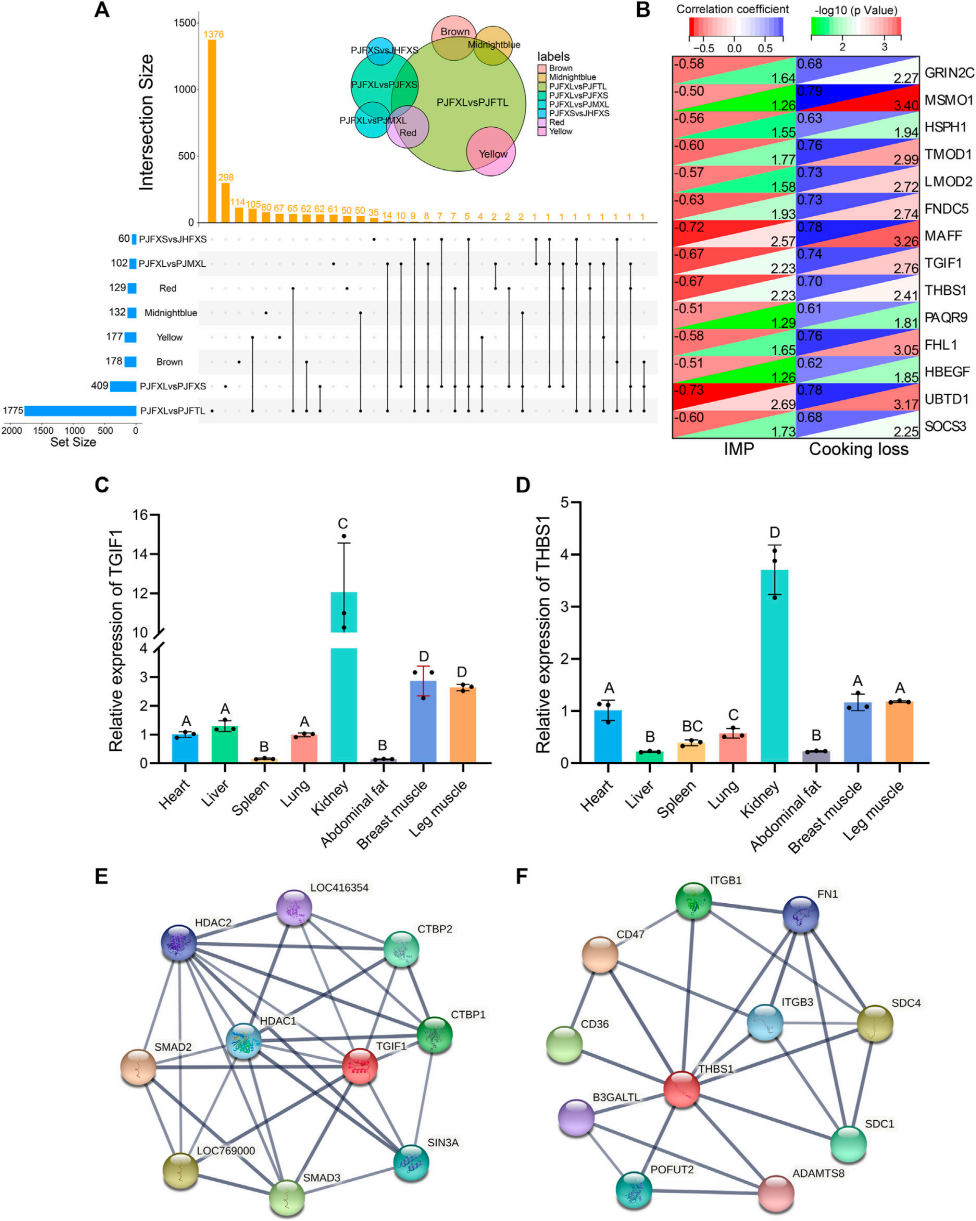
Functional gene identification. **(A)** Upset Venn diagram of hub genes and DEGs in four important modules. **(B)** Correlation of 14 DEGs with IMP and cooking loss (The value in the upper left corner of the figure is the correlation coefficient, and the value in the lower right corner is −log10 (*p*-value)). Tissue expression of **(C)** TGIF1 and **(D)** THBS1 in Jingyuan chickens. The protein interaction network of **(E)** TGIF1 and **(F)** THBS1 (Network nodes represent proteins: splice isoforms or post-translational modifications are collapsed, i.e., each node represents all the proteins produced by a single, protein-coding gene locus. Edges represent protein-protein associations: associations are meant to be specific and meaningful, i.e., proteins jointly contribute to a shared function).

Association analysis of hub genes in the four significant modules with intergroup DEGs was performed and 14 intersecting genes *GRIN2C, SOCS3, MSMO1, HSPH1, TMOD1, LMOD2, FNDC5, MAFF, TGIF1, THBS1, PAQR9, FHL1, HBEGF*, and *UBTD1* were common to at least one module and two differential comparison groups (Figure 7A). These 14 genes were positively correlated with cooking loss (*p* < 0.05) and 11 were negatively correlated with IMP (*p* < 0.05) (Figure 7B). The TGF-β signaling pathway genes *TGIF1* and *THBS1* were strongly correlated with both IMP and cooking loss (*p* < 0.05) and were expressed at high levels in the breast and leg muscles of Jingyuan chickens (Figures 7C, D). Protein interaction network analysis showed that *TGIF1* and *THBS1* interacted with nine and ten proteins, respectively (Figures 7E, F), with a transcription regulator activity function. Taken together, *TGIF1* and *THBS1* may regulate the expression of key enzymes involved in IMP anabolism, either directly or indirectly, through muscle biogenesis-related processes.

## 4 Discussion

The economic value of poultry meat is directly related to its quality. Chicken meat quality may vary greatly depending on the body part, feeding method, sex, and breed. A previous study found that the IMP content in the breast muscle of a caged Jingyuan hen and rooster was significantly higher than that in the leg muscle, and the IMP content in the hen was higher than that in the rooster; however, the difference was not significant (Zhang et al., 2020). The IMP content of free-range hens is significantly higher than that of caged hens under different feeding patterns (Wang et al., 2022), probably because of the increase in muscle ATP content resulting from increased activity and enhanced IMP synthesis. Moreover, there were significant differences in IMP content between breeds in the muscles of Jingyuan and Pudong chickens (Zhang et al., 2021). These results are consistent with those of previous studies (Liu et al., 1980; Zhang et al., 2014; Liu et al., 2018; Wang et al., 2018). The reason for these differences may be due to differences in the expression of key enzymes involved in IMP synthesis. Although preliminary studies have explored the gene networks regulating IMP deposition, most have focused only on single-gene expression aspects (Hu et al., 2015; Zhang et al., 2015.; Zhang T. et al., 2018). There is a paucity of relevant studies revealing the molecular mechanisms underlying IMP-specific deposition in chicken muscles from multiple perspectives.

We explored the genetic factors affecting muscle IMP-specific deposition in Jingyuan chickens from several perspectives. We analyzed the transcriptome profiles of Jingyuan chickens for different parts, feeding methods, sexes, and compared them with Pudong chickens to identify key genes associated with muscle IMP. In total, 1,775 DEGs were identified between the breast and leg muscles of caged Jingyuan hens, 409 DEGs in the breast muscles of caged and free-range hens, 102 DEGs in the breast muscles of caged hens and roosters, and 60 DEGs in the breast muscles of free-range Jingyuan and Pudong chickens. Further exploration of the specific biological functions of the DEGs revealed that the DEGs of the four comparison groups could participate in multiple biological processes of muscle development, including several metabolic processes such as energy metabolism and lipid anabolism. It has been shown that the glycolysis/gluconeogenesis pathway is the main form of energy metabolism in the organism, and the energy generated by its metabolic processes plays a critical role in ensuring cell survival and growth. PPARγ can bind and activate the transcriptional activity of pyruvate kinase M in the form of transcription factors, which regulate glycolysis/gluconeogenesis processes (Panasyuk et al., 2012). The PPAR signaling pathway regulates lipid metabolism (Moreno et al., 2010; Cui et al., 2012). Additionally, FoxO is a key pathway involved in gluconeogenesis (Zhang X. et al., 2018). Cytokine-cytokine receptor interactions promote focal adhesions (Wang et al., 2021). Focal adhesions play a vital role in skeletal muscle development and are a signaling center for cell growth and differentiation (Sastry and Burridge, 2000). These results suggest that intergroup DEGs may be involved in the anabolic processes of IMP in the form of energy metabolism, key molecular activities, and direct or indirect participation in muscle quality regulation.

Traditional transcriptome sequencing methods cannot distinguish confounding factors among multiple groups (Barabasi and Oltvai, 2004; Ghaemi et al., 2019). Many DEGs can be identified by transcriptome sequencing; however, reliable evidence to elucidate the gene network associated with meat quality phenotypes is lacking. WGCNA is an effective data-mining method that can cluster massive gene sets into co-expression modules based on gene expression patterns (Zhao et al., 2010). Co-expression modules and key genes that perform biological functions were identified through association analysis with phenotypic traits. In this study, we constructed 11 functional co-expression modules, among which the brown, midnight blue, red, and yellow modules showed a strong correlation with both IMP and cooking loss.

GO and KEGG enrichment analyses explored possible mechanisms by which functional genes regulate muscle development and IMP deposition. GO enrichment analysis revealed significantly enriched functional terms for protein and kinase activity regulation, molecular transduction and metabolism, growth factor activity, transcriptional regulation, and skeletal muscle development. IMP is mainly produced by the degradation of ATP in muscles. Therefore, muscle development is closely related to IMP anabolic processes. IMP is also known as a hypoxanthine nucleotide and the purine metabolic pathway regulates its anabolism. Pyruvate kinase M (PKM), a key gene in the purine metabolic pathway, regulates adenine ribonucleotide biosynthesis (IMP => ADP, ATP) and guanine ribonucleotide biosynthesis (IMP => GDP, GTP) (Yu et al., 2022), as well as a variety of amino acid-assisted regulations in this process. At the same time, PKM is also the key kinase regulating the conversion of glucose to pyruvate in the glycolysis pathway, which has many functions, such as anabolism, cell proliferation, and aerobic glycolysis (Deyle et al., 2012). These results suggest that glycolysis/gluconeogenesis, arginine, proline, and pyruvate metabolism play important roles in IMP-specific deposition processes.

Analysis of the hub genes of the functional modules revealed that *PYCR1*, *SMOX*, and *ACSS2* were the most important genes. Pyrroline-5-carboxylate reductase 1 (*PYCR1*) is a precursor of l-proline synthesis and is involved in the regulation of proline biosynthesis (Alaqbi et al., 2022). A key step in proline biosynthesis is the reduction of Δ^1^-pyrroline-5-carboxylate (P5C) to proline, which is catalyzed by P5C reductase (PYCR). Studies have shown that proline biosynthesis is key to sustaining protein synthesis and supporting mitochondrial function, redox balance, signaling, and nucleotide biosynthesis (Burke et al., 2020; D’Aniello et al., 2020; Ding et al., 2020; Tran et al., 2021). Spermine oxidase (*SMOX*) is a multifunctional enzyme that controls polyamine metabolism, plays an important role in muscle differentiation, and maintains muscle fiber size and skeletal muscle mass (Cervelli et al., 2018; Reinoso-Sánchez et al., 2020). Vertebrate *SMOX* is a flavoprotein that specifically oxidizes the natural substrate spermine, with the production of spermidine, hydrogen peroxide (H_2_O_2_), and the aldehyde 3-aminopropanal (Polticelli et al., 2012; Cervelli et al., 2013). H_2_O_2_ plays a critical regulatory role in skeletal muscle function. H_2_O_2_ levels from low to moderate are critical for cell signaling and regulation of gene expression; they act as signals for cell adaptation and are necessary for muscle growth (Powers and Jackson, 2008; Sies, 2017).

Acyl-CoA synthetase short-chain family member 2 (*ACSS2*) is a conserved nucleocytosolic enzyme that affects lipid synthesis and metabolism by selectively regulating genes related to lipid metabolism (Huang et al., 2018). The basic function of the ACS family of enzymes is to convert acetate and coenzyme A (CoA) into acetyl-CoA in an ATP-dependent manner. It was found that *ACSS2* encodes acetyl coenzyme A synthetase, and was expressed at approximately 2-fold higher levels in subcutaneous fat than in intramuscular fat, consistent with a relative preference for acetate in the subcutaneous fat depot (Hudson et al., 2020). STRING database exploration revealed that *ACSS2* has functions similar to those of adipogenesis-related genes (*ACACA*, *ACOT12*, *SIRT3*, and aldehyde dehydrogenase family members) and can regulate acetyl-CoA metabolism, fatty acid biosynthesis, and adipocyte differentiation. Consistent with the results of Hu et al. (2010), *ACACA* was identified as a key factor in adipogenesis and transport, and played a crucial role in the weight variability of abdominal adipose tissue in growing chickens. IMP deposition is a product of the interaction of multiple biological processes mediated by a complex network of gene regulation in muscles. Although there is no direct evidence that *PYCR1*, *SMOX*, and *ACSS2* are associated with muscle IMP, the association analysis of transcriptome DEGs and co-expression functional modules suggests that *PYCR1*, *SMOX*, and *ACSS2*, which are regulated by the glycolysis/gluconeogenesis, arginine and proline metabolism, and pyruvate metabolism pathways, may be potential candidate genes for regulating IMP deposition.

Based on the combined analysis of the transcriptome and co-expression module, TGF-β signaling pathway-regulated TGIF1 and THBS1 were strongly correlated with both IMP and cooking loss and showed high expression levels in the breast and leg muscle tissues of Jingyuan chickens. TGF-β is a multifunctional secreted protein belonging to the transforming growth factor (TGF) superfamily. TGF-β can inhibit myoblast differentiation during the myoblast state transition by promoting *MYOD* degradation and inhibiting myogenin expression (Schabort et al., 2009). During myogenic differentiation, core proteoglycans in the extracellular matrix can competitively bind TGF-β, resulting in reduced TGF-β binding ability to the receptors TGF-β R1 and TGF-β R2, affecting satellite cell differentiation (Droguett et al., 2006). TGF-β and Wnt pathways also interact to regulate skeletal muscle growth and development (Biressi et al., 2014). Furthermore, the TGF-β signaling pathway typically inhibits adipocyte differentiation (Du et al., 2013). A previous study found that TGF-β3 stimulates adipocyte progenitor proliferation in white adipose tissue, which is related to glucose metabolism (Petrus et al., 2018). This is consistent with our findings that the TGF-β signaling pathway, which regulates multiple molecular activities and energy metabolism, may be a key regulator of muscle development and IMP deposition in Jingyuan chickens.

TGFB-induced factor homeobox 1 (*TGIF1*) is a multifunctional protein that represses TGF-β-activated transcription by interacting with Smad2-Smad4 complexes (Guca et al., 2018). By analyzing open chromatin regions and transcription factor-binding sites in porcine dorsal longissimus muscle, *TGIF1* was identified as a possible transcription factor that affects muscle growth and development (Miao et al., 2021). The *TGIF1* gene was identified in a gene module related to the growth and development of pigeon skeletal muscle, which has a high degree of connectivity and is a hub gene in development-specific modules (Ding et al., 2021). In addition, *TGIF1* is involved in regulating lipid metabolic processes; knockout of *TGIF1* in mice increases the accumulation of intrahepatic lipids and serum levels of cholesterol (Pramfalk et al., 2014), and *TGIF1* represses *ACAT2* and *NPC1L1* (Parini et al., 2018). Thrombospondin-1 (*THBS1*), a multidomain calcium-binding glycoprotein (Adams and Lawler, 2011), is highly expressed during muscle development following injury (Stenina et al., 2003). *THBS1* is also elevated in obesity and is an adipocyte-derived cytokine (adipokine) (Varma et al., 2008). STRING database exploration revealed that *THBS1* functions in cell adhesion, fibronectin binding, and cell surface and is also regulated by KEGG pathways such as extracellular matrix receptor interaction, focal adhesion, and other types of O-glycan biosynthesis. Freedman et al. (2018) found that the extracellular matrix plays an important role in multiple cell proliferation and differentiation processes and can also interact with focal adhesion signaling to participate in the growth and differentiation of skeletal muscle cells (Sastry and Burridge, 2000; Romer et al., 2006). These studies suggest that *TGIF1* and *THBS1* regulate the synthesis and metabolism of IMP in muscles directly or indirectly through biological processes related to myogenesis.

## 5 Conclusion

Transcriptome analysis of different muscle tissues, feeding methods, and sexes of Jingyuan chickens, and comparison with other breeds, combined with differential expression analysis and WGCNA, helped to identify multiple potential candidate genes regulating muscle IMP deposition, including *PYCR1*, *SMOX*, *ACSS2*, *TGIF1*, and *THBS1*. Glycolysis/gluconeogenesis, TGF-β signaling pathway, and other multiple functional mechanisms are important in IMP-specific deposition. This study explored the genes regulating muscle IMP synthesis and metabolism in Jingyuan chickens from multiple perspectives, providing an important theoretical basis for the improvement of meat quality and molecular breeding of chickens.

## Data Availability

The authors declare that the data supporting the findings of this study are available within the article and its Supplementary Material. All the raw sequences have been deposited in the NCBI database Sequence Read Archive with the accession numbers PRJNA957235 (https://dataview.ncbi.nlm.nih.gov/object/PRJNA957235).
