# Supplementary material for: Transcriptome and co-expression network analysis reveals the molecular mechanism of inosine monophosphate-specific deposition in chicken muscle
Source: Front Physiol. 2023 May 17;14:1199311. doi: 10.3389/fphys.2023.1199311 (PMC10229883; doi:10.3389/fphys.2023.1199311)
Supplement: Supplementary file 1 [file DataSheet1.ZIP › Figure S1.docx]

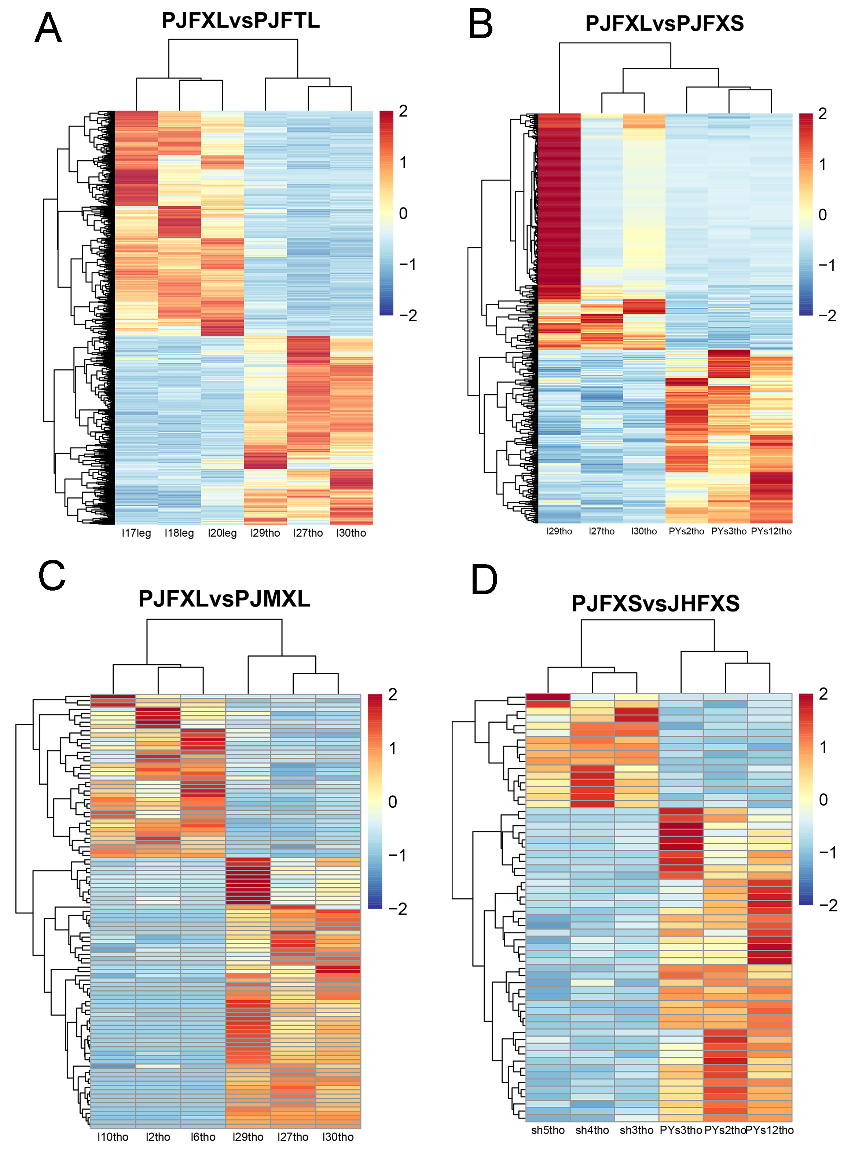


**Supplementary Figure 1.** Hierarchical clustering heatmap of DEGs in (A) PJFXL vs. PJFTL, (B) PJFXL vs. PJFXS, (C) PJFXL vs. PJMXL, and (D) PJFXS vs. JHFXS comparison groups.
